# Keeping the goal in mind: Prefrontal contributions to spatial navigation

**DOI:** 10.1016/j.neuropsychologia.2008.01.028

**Published:** 2008-06

**Authors:** Hugo J. Spiers

**Affiliations:** Institute of Behavioural Neuroscience, Department of Psychology, University College London, 26 Bedford Way, London WC1H 0AP, United Kingdom

**Keywords:** Wayfinding, Spatial memory, Inhibition, Planning, Orbitofrontal

On his voyage home, Odysseus is nearly seduced to his death by the singing of the sirens. Odysseus's crew, their ears plugged with wax, thankfully do not hear his desperate pleas to change course. While such trials are not a feature of our daily travels, a variety of distractions can send us off course. In this issue, Ciaramelli (2008) reports a patient who repeatedly suffers from this affliction, being lured away to unintended locations. The study provides new insights into the neural basis of spatial navigation.

Our capacity for navigation is thought to rely on a distributed network of brain regions, which include the hippocampus, parahippocampus, retrosplenial cortex, parietal cortex, and caudate nucleus (see e.g. Aguirre & D’Esposito, 1999; Spiers & Maguire, 2007a). Each region is thought to serve a different function of our navigation machinery, such as representing a map of large-scale space, converting the map to egocentric space, or representing our current viewpoint. However, the neural substrate of one particular function remains a mystery. How does the brain represent spatial goal locations or guide navigation to them? Several lines of evidence suggest this may be the preserve of the prefrontal cortex.

The prefrontal cortex has long been associated with behavioural flexibility, working memory and planning; functions important for achieving goals (Fuster, 1989; Luria, 1969; Passingham, 1993). It also integrates highly processed information important for guiding goal-directed behaviour (Pandya & Barnes, 1987). Several functional neuroimaging studies have revealed increased activity in prefrontal areas during spatial navigation tasks (Gron, Wunderlich, Spitzer, Tomczak, & Riepe, 2000; Hartley, Maguire, Spiers, & Burgess, 2003; Maguire et al., 1998; Yoshida & Ishii, 2006), one directly linking prefrontal activity to goal processing (Spiers & Maguire, 2006, 2007b). In rodents, medial and orbital prefrontal lesions have been found to impair certain aspects of navigation (Lacroix, White, & Feldon, 2002; Vafaei & Rashidy-Pour, 2004), and recently cells in these regions have been found to code spatial information about goals (Feierstein, Quirk, Uchida, Sosulski, & Mainen, 2006; Hok, Save, Lenck-Santini, & Poucet, 2005). However, it remains uncertain whether the human prefrontal cortex is *necessary* for navigation.

In a recent article in Neuropsychologia new evidence has emerged suggesting that the ventromedial prefrontal cortex (vmPFC) is required for navigation (Ciaramelli, 2008). Ciaramelli (2008) tested a patient with bilateral damage to the ventromedial prefrontal and rostral anterior cingulate cortices whose central complaint, following recovery, was of way-finding difficulties. Despite good topographical knowledge of his hometown he performed very poorly, compared to healthy controls, when asked to describe a set of routes between locations in the town. However, his performance improved substantially when he was given the name of his destination or a cue to rehearse the destination at regular intervals. No such improvement occurred when a visual stimulus was presented at similar intervals. Thus, it appears the vmPFC is necessary for navigation and its role may be to maintain the goal destination in working memory (Ciaramelli, 2008). That the problem lies with working memory is further supported by the patient's generalised deficits on standardized tasks requiring working memory.

However, deficits elicited by laboratory tasks do not always predict deficits in the ‘real world’ (Habib & Sirigu, 1987; Kapur & Pearson, 1983; Maguire, Burke, Phillips, & Staunton, 1996; Spiers, Burgess, Hartley, Vargha-Khadem, & O’Keefe, 2001). Indeed, a cue to rehearse the destination might not be as beneficial during navigation of a complex and bustling town. Appropriately, the patient's ability to actively navigate in the town was assessed under similar cue conditions to the laboratory task, with a strikingly similar pattern of results.

Further insight into this patient's problem was generated by an analysis of the errors made in relation to several factors, including familiarity, route length, number of turns, etc. (Ciaramelli, 2008). Of these factors, only familiarity was found to be associated with the number of errors. While successful routes were rated more familiar, surprisingly, the reverse was true of highly familiar locations on the route. Intriguingly, two-thirds of error trials involved the route ending at one of a number of personally familiar locations, each associated with the patient's previous work or hobbies. According to Ciaramelli (2008) these locations, being highly salient, acted as “attractor” locations, luring the patient away from his true goal. Thus, the vmPFC may be necessary not only to maintain the goal in memory, but also to suppress irrelevant information.

These findings agree well with those of a recent neuroimaging study exploring the brain activity of London taxi drivers as they navigated a highly accurate virtual simulation of London (UK) (Spiers & Maguire, 2006, 2007b). In this study, the relationship between the subject's thoughts during navigation and their brain activity was examined using a retrospective verbal report protocol. Immediately post-scan, subjects watched a video replay of their performance and reported what they had been thinking while they were doing the task in the scanner. Of the wide variety of thoughts reported, the most frequent concerned thinking about the goal and the route to it (see Spiers & Maguire, in press, for full details). These were associated with increased activity in anterior BA10 and in a medial prefrontal region overlapping with the dorsal extent of the lesion of the patient described by Ciaramelli (2008). Given the latest findings, it could be argued the medial region is involved in maintaining goal representation while B10 may be important for manipulating information for planning (Koechlin, Basso, Pietrini, Panzer, & Grafman, 1999). Further evidence that cells in the medial prefrontal region might monitor spatial goals comes from the finding that activity in a region on the border of BA9 and BA32 correlated with proximity to the goal during navigation (Spiers & Maguire, 2007b). A more ventral medial prefrontal region was activated when subjects listened to navigationally irrelevant comments of customers whilst navigating (Spiers & Maguire, 2006). Ciaramelli (2008) suggests this may have been related to the mental rehearsal or ‘energizing’ of the actual goals. An alternative, related, interpretation is that the region is activated by the requirement to suppress processing of the new information in posterior cortical regions.

Ciaramelli (2008) argues that the patient's deficit may arise from three related difficulties. These are: (a) an inability to actively maintain the goal in working memory, (b) a reversal-learning problem, and (c) a loss of the grammar that prescribes the transitions between attractor states in cortical networks. The last problem would make it difficult for the patient to avoid personally familiar locations, as these would be represented by strong attractors. A view favoured here, in line with other authors (Burgess, Veitch, Costello, & Shallice, 2000; Shallice & Burgess, 1991), is that the deficit can more readily be summarised as a failure to maintain the *intention* to reach the destination in working memory and a reduced suppression of previously learned information (in this case routes). A similar lack of suppression may underlie the reversal-learning deficit seen in such patients (Fellows & Farah, 2003), rather than vice versa.

Most new research generates more questions than answers, as is the case here. Along with replicating the findings in other similar patients, a number of questions remain to be answered. Can the patient learn routes in an unfamiliar environment? At what point in the route is the intention lost? Given that medial prefrontal activity is correlated with goal proximity (Spiers & Maguire, 2007b), might the patient be unable to make use of novel shortcuts or reach the goal after a detour? Which region calculates proximity or directional information to goals? Though there is much still to be explored, the results from Ciaramelli (2008) add to our understanding of the neural processes supporting navigation. Given this new insight, when your mind next drifts and you arrive at a familiar but unintended destination, you will know which part of your neuroanatomy to blame.
